# RfaH is essential for virulence and adaptive responses in *Yersinia pseudotuberculosis* infection

**DOI:** 10.1128/mbio.02122-25

**Published:** 2025-09-29

**Authors:** Joram Kiriga Waititu, Kristina Nilsson, Gerald Larrouy-Maumus, Tiago R. D. Costa, Kemal Avican

**Affiliations:** 1 Department of Molecular Biology, Umeå University 8075, Umeå, Sweden; 2 Integrated Science Lab (IceLab), Umeå University 8075, Umeå, Sweden; 3 Umeå Centre for Microbial Research (UCMR), Umeå University 8075, Umeå, Sweden; 4 Centre for Bacterial Resistance Biology, Imperial College London 4615, London, United Kingdom; 5 Department of Life Sciences, Imperial College London 98455, London, United Kingdom; Cornell University College of Veterinary Medicine, Ithaca, New York, USA

**Keywords:** RfaH, infection, RNA-seq, *Yersinia*, cell surface

## Abstract

We previously suggested that increased expression of the gene encoding transcriptional antiterminator RfaH during *Yersinia pseudotuberculosis* transcriptional reprogramming is necessary for adapting to persistent infection. In this study, we examined the role of RfaH in virulence and bacterial physiology under infection-relevant stress conditions, and identified genes differentially regulated in the absence of RfaH in *Y. pseudotuberculosis*. We employed a mouse infection model and phenotypic assays to test RfaH’s role in virulence and physiology, as well as RNA sequencing, including O-antigen biosynthesis-deficient strains. Our findings demonstrate that loss of RfaH significantly attenuates virulence, reducing the capacity of *Y. pseudotuberculosis* to establish persistent infection. RfaH expression is increased during the stationary growth phase and under various stress conditions, such as high osmolarity and bile salts, which are known to induce envelope stress. Functional assays revealed that the Δ*rfaH* strain displayed defects in motility and increased clumping, indicating altered surface properties affecting motility. Moreover, transcriptomic profiling of the Δ*rfaH* strain revealed a specific RfaH-dependent gene set after filtering out genes affected by O-antigen-related mutations, thereby minimizing confounding effects from surface structure alterations. These results suggest that RfaH influences a broader set of virulence and adaptation pathways beyond O-antigen regulation. Collectively, our findings suggest that RfaH is essential for the virulence and adaptive capacity of *Y. pseudotuberculosis* to colonize the host. This study provides insights into regulatory mechanisms that facilitate bacterial survival in hostile environments and highlights the importance of RfaH and its regulatory targets in the pathogenesis of *Y. pseudotuberculosis*.

**IMPORTANCE:**

For bacterial pathogens to establish infection and persist in the host, they must adapt to harsh environments and fine-tune gene expression accordingly. The transcriptional antiterminator RfaH plays a pivotal role in regulating key genes essential for adaptation and virulence in *Y. pseudotuberculosis*. In this study, we explored the function of RfaH in bacterial physiology, stress responses, and infection dynamics. Using a mouse infection model, we found that loss of RfaH significantly reduced virulence and impaired the pathogen’s ability to establish persistent infection. Notably, RfaH expression increased under stress conditions, such as high osmolarity and temperature, underscoring its role in bacterial adaptation. On the other hand, the absence of RfaH led to motility defects and enhanced bacterial aggregation, suggesting alterations in surface properties. Transcriptomic analysis revealed that RfaH influences a broader set of genes beyond the O-antigen biosynthesis operon, including virulence factors critical for host adaptation. Overall, our findings establish RfaH as a key regulator of *Y. pseudotuberculosis* virulence, shedding light on the molecular mechanisms that enable bacterial survival in challenging environments.

## INTRODUCTION

The genus *Yersiniae*, in the family *Enterobacteriaceae*, consists of 11 species, of which *Yersinia pseudotuberculosis*, *Y. enterocolitica*, and *Y. pestis* are known to cause diseases in mammals (1–3). *Y. pestis*, transmitted by fleas, is the causative agent of plague (4), while both *Y. pseudotuberculosis* and *Y. enterocolitica* are enteric pathogens that are typically acquired by ingestion of contaminated food or water, leading primarily to self-limiting conditions such as adenitis, enteritis, diarrhea, and ileitis (2, 5, 6). High-dose *Y. pseudotuberculosis* infection in rodents, such as guinea pigs and mouse models, results in systemic infection due to translocation from the intestinal tract to the spleen and liver (7). The pathogenicity of all three *Yersinia* species is largely dependent on the type III secretion system and its related virulence substrate proteins, known as *Yersinia* outer proteins, which are encoded on a 70-kb virulence plasmid (8, 9). Low-dose *Y. pseudotuberculosis* oral infections cause chronic infection in mouse cecal tissue without signs of disease (10). This model of *Y. pseudotuberculosis* persistent infection offers insight into bacterial mechanisms of importance for the initiation and maintenance of persistent infections. We have previously explored the nature of *Y. pseudotuberculosis* persistent infection by profiling transcriptional changes from the early to the later persistent phase of infection (11). The study identified potentially significant key players during persistence, which primarily reflected the environmental conditions the pathogen encountered in the cecal lymphoid follicles. A set of global transcriptional regulators, such as the Crp/CsrA/RovA cascade, regulated gene expression, allowing *Y. pseudotuberculosis* to adapt to long-term residence in the host (11). Notably, expression of the gene encoding the transcriptional regulator RfaH was significantly enhanced during the persistent state of infection but was not investigated further (11).

RfaH is a transcriptional regulator belonging to the NusG family of proteins (12). It enables the complete transcription of long operons by preventing Rho-dependent premature termination (13, 14). The specificity of RfaH for its target genes depends upon a 12-nucleotide conserved regulatory site called the operon polarity suppressor (*ops*), which is typically located at upstream regions of the operons regulated by RfaH (15, 16). RfaH prevents early transcriptional termination and enhances transcriptional elongation, ensuring, for example, lipopolysaccharide (LPS) biosynthesis, as shown for many bacteria (17). In *Escherichia coli*, RfaH acts as a specialized transcription-translation coupling factor that is recruited to RNA polymerase (RNAP) at the *ops* site, where it bridges RNAP and the lead ribosome via ribosomal protein S10, thereby accelerating transcription elongation rates and blocking Rho-dependent termination (18, 19). This coupling mechanism distinguishes RfaH from NusG, as the activity of the former is restricted to operons containing the *ops* element (18), and such coupling also contributes to regulatory mechanisms like transcription attenuation and riboswitch control (18, 20, 21). In addition, RfaH has been linked to various roles, including initiation of translation by interacting with the 30S ribosome (12), regulation of operons involved in capsular biosynthesis (22), hemin uptake components (23), and production of toxins like hemolysins and cytotoxic necrotizing factor (24). Indeed, survival of *Vibrio vulnificus* in serum is dependent on RfaH (25). RfaH has also been implicated in the pathogenesis of various pathogens, where its loss leads to attenuation in virulence of the *Salmonella* (23), *E. coli* (26), and *Klebsiella* (27). In *Y. pseudotuberculosis,* RfaH has been reported to contribute to resistance against antimicrobial chemokines and survival during mouse infections (28). However, the precise mechanisms by which RfaH enhances the expression of these components remain incompletely understood. Despite significant scientific advances in understanding the RfaH mechanism in various pathogens, knowledge about its molecular mechanisms in microbial pathogenesis, particularly *Y. pseudotuberculosis,* remains limited (29). In this study, we conducted phenotypic assays to dissect RfaH-dependent changes during bacterial growth and the establishment of infection in a mouse model. Furthermore, we extended our understanding of RfaH’s role in the pathogenesis of *Y. pseudotuberculosis* through transcriptomic analysis by performing transcriptomic profiling of wt, *rfaH* deletion mutants, and various RfaH-related mutant strains of *Y. pseudotuberculosis* at both environmental (26°C) and host body (37°C) temperatures to identify RfaH-regulated genes. We identified common and mutation-specific genes and pathways, shedding light on the potential molecular mechanisms of RfaH in persistent *Y. pseudotuberculosis* infections. Our findings provide a deeper understanding of RfaH’s role in *Y. pseudotuberculosis* virulence mechanisms and offer a valuable molecular reference for future studies to understand persistent infections caused by this pathogen.

## RESULTS

### RfaH expression is growth phase-dependent and can be induced by envelope stress

**Fig 1:**
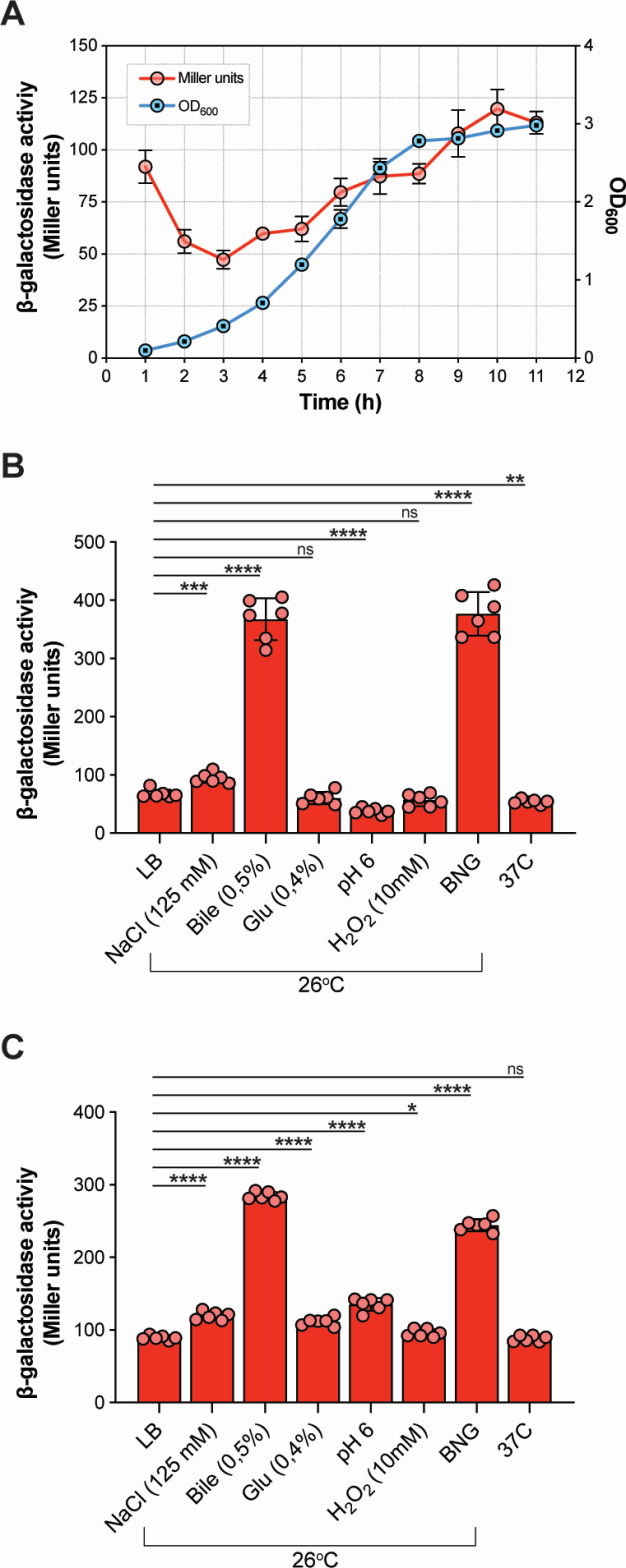
RfaH expressions increased during the stationary phase and under infection-relevant stress conditions. (**A**) The β-galactosidase activity of the RfaH and LacZ translational fusion construct reflected in the hourly increase in optical density at 600 nm (OD_600_) during bacterial growth at 26°C. (**B and C**) The β-galactosidase activity of RfaH and LacZ translational fusion construct in bacterial cultures at exponential phase (**B**) and stationary phase (**C**), after treatment with infection-relevant stressors: 125 mM NaCl, 0.5% bile salts, 0.4% glucose, pH 6, 10 mM H_2_O_2_, and a combination of 125 mM NaCl, 0.5% bile salts, and 0.4% glucose (BNG). Six independent biological replicates were used in panels A, B, and C. In panel A, dots represent the mean values of the six biological replicates, with error bars indicating standard deviations. In panels B and C, the bars represent the mean of six biological replicates (also shown as individual dots). Statistical significance was determined using an unpaired *t*-test, with Luria broth medium as the control. Asterisks indicate *P*-values as *<0.05, **<0.01, ***<0.0005, and ****<0.0001 and ns (not significant) as >0.05.

Given that the *in vivo* transcriptome profile of persistent *Y. pseudotuberculosis* resembles that of bacteria in the stationary phase (11), we investigated whether RfaH protein expression in *Y. pseudotuberculosis* is dependent on the bacterial growth phase. To do this, we generated an RfaH-LacZ translational fusion construct where the RfaH regulatory region, including the promoter region, ribosome binding site, and the coding sequence of the first 13 amino acids, was fused to the *lacZ* coding sequence without a start codon and measured the β-galactosidase activity of the construct. As a result, the expression of RfaH increased progressively over time and peaked during the late stationary phase (Fig. 1A), a pattern consistent with that observed in *Salmonella enterica* serovar Typhimurium (30, 31). This finding provided a starting point for exploring environmental cues that may influence RfaH production. Additionally, under *in vitro* conditions mimicking the host environments, we investigated the impact of specific environmental factors such as high osmolarity, bile salts, glucose, low pH, oxidative stress, temperature, and a combination of high osmolarity, bile salts, and glucose (BNG) on RfaH expression during both exponential and late stationary growth phases. To determine whether *in vivo*-relevant stress conditions can influence RfaH expression during exponential growth, when its baseline expression is relatively low, we exposed exponentially growing bacteria (OD₆₀₀ ≈ 0.4–0.5) to various stress conditions for 1 hour. High osmolarity, bile salts, and BNG significantly induced RfaH expression even at this early growth stage, with bile salts and BNG causing particularly dramatic increases (Fig. 1B). In contrast, low pH and 37°C slightly reduced RfaH expression, while glucose supplementation and oxidative stress had negligible effects (Fig. 1B). To test whether these conditions could further enhance RfaH levels during a phase when the expression is already elevated, we repeated the experiments in stationary-phase cultures. All tested conditions led to significant increases in RfaH expression, with bile salts and BNG again producing the most pronounced induction (Fig. 1C). Notably, unlike in the exponential phase, RfaH expression was not reduced at 37°C in stationary-phase cells. Thus, the host-associated stress conditions stimulate RfaH expression during both exponential and stationary growth phases, indicating that this regulatory protein may play a role in the pathogen’s adaptability to hostile environments in the host. The dramatic increase of RfaH expression under high osmolarity, bile salts, and BNG combinations, which are known to induce envelope stress (32, 33), suggests that RfaH expression might be induced by envelope stress.

### Lack of RfaH is associated with bacterial aggregation and reduced motility

**Fig 2:**
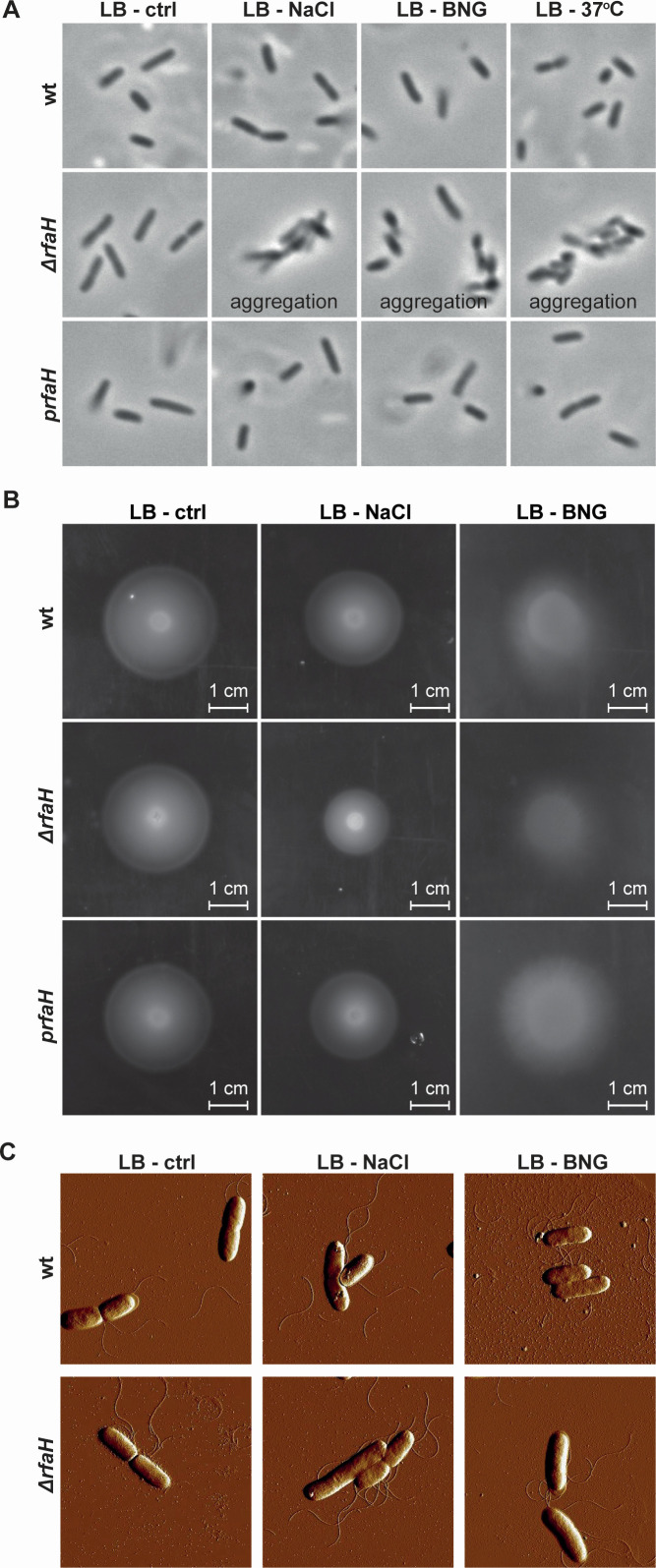
Loss of *rfaH* reduces motility despite the presence of flagella under stress conditions. (**A**) Phase-contrast microscopy images of wt, Δ*rfaH,* and Δ*rfaH* complemented with in *trans rfaH* expression (*prfaH*) strains under various conditions: control (26°C), 125 mM NaCl, a combination of 125 mM NaCl, 0.5% bile salts, and 0.4% glucose (BNG), and at 37°C. (**B**) Motility profile of wt, Δ*rfaH,* and *prfaH* strains under control, 125 mM NaCl, and BNG conditions at 26°C. (**C**) Atomic force microscopy images of the flagella from the wt and Δ*rfaH* strains under control, 125 mM NaCl, and BNG conditions at 26°C.

To investigate the impact of *rfaH* deletion, we performed phenotypic assays and assessed bacterial morphology using both light and atomic force microscopy. Assays were conducted under conditions with high RfaH expression, as well as under a control condition at 26°C. The Δ*rfaH* strain aggregated after being subjected to NaCl, BNG, and temperature (37°C for 2 hours), suggesting that RfaH influences bacterial surface structures (Fig. 2A). However, the expression of *rfaH* in *trans* complemented the aggregation phenotype (Fig. 2A). To further assess motility, we performed soft agar motility assays at 26°C using both wt and Δ*rfaH* strains, following exposure to NaCl and BNG. This temperature was chosen since *Y. pseudotuberculosis* is non-motile at 37°C *in vitro,* and previous transcriptomic profiling showed that specifically during persistent infection, flagellar gene expression partially resumes and the global transcriptional profile resembles that of bacteria grown at 26°C (11). Under high osmolarity and BNG treatment, the Δ*rfaH* strain displayed reduced motility, and this defect was rescued by complementation of *rfaH* in *trans* (Fig. 2B). To determine whether the motility defects were due to structural changes in flagella, we visualized both untreated and stress-treated bacterial cells using atomic force microscopy. Despite the loss of motility, the Δ*rfaH* strain retained flagella in both NaCl and BNG-supplemented media (Fig. 2C). These findings suggest that the reduced motility is not due to flagella absence but may be due to the involvement of RfaH in regulating surface structures, potentially through its influence on cell aggregation or chemotaxis signaling pathways.

### Defective O-antigen biosynthesis contributes to RfaH-dependent phenotypic changes

**Fig 3:**
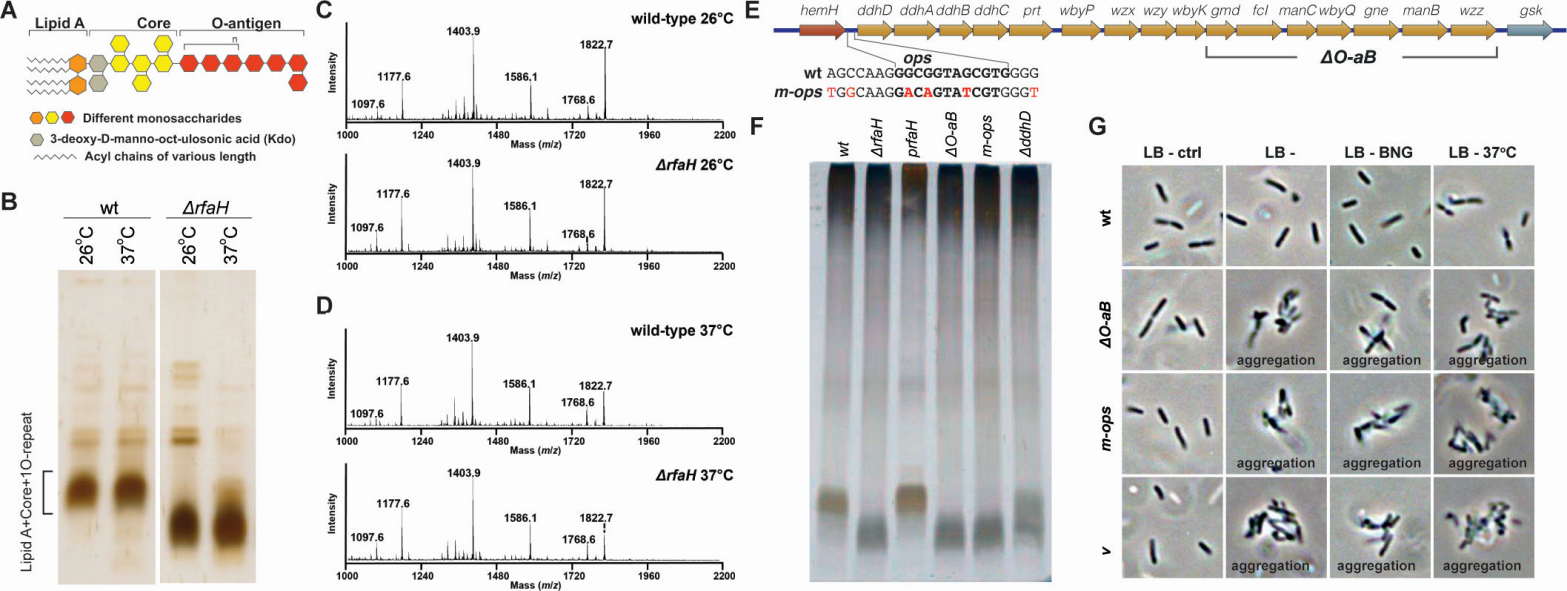
RfaH-related phenotypic changes are indirectly influenced by defective O-antigen biosynthesis. (**A**) Composition of LPS in *Y. pseudotuberculosis*. (**B**) LPS profiles of wt and Δ*rfaH* mutant strains were analyzed at 26°C and 37°C via 15% SDS-PAGE with silver staining. (**C and D**) Representative MALDI-TOF mass spectra of lipid-A from wt and Δ*rfaH* (**C**) at 26°C and (**D**) at 37°C. (**E**) Schematic of the O-antigen biosynthesis operon, highlighting the *ops* sequence located upstream. Nucleotide substitutions introduced to generate the *m-ops* mutant are marked in red. The array of seven genes on the distal part of the operon is shown for the Δ*O-aB* deletion mutant. (**F**) LPS profiles of wt, Δ*rfaH*, and other O-antigen biosynthesis operon mutants and the Δ*rfaH* complementation strain were analyzed at 26°C via 15% SDS-PAGE with silver staining. (**G**) Phase-contrast microscopy images of multiple mutants with defective O-antigen biosynthesis.

Given that RfaH is known to regulate LPS in *Y. pseudotuberculosis* (28), we hypothesized that the aggregation observed in the Δ*rfaH* mutant strain might be due to defective LPS. A likely explanation is that shortened LPS exposes underlying outer membrane adhesins such as autotransporters like Ag43 that are normally masked by intact LPS. Exposure to these adhesins has been shown to enhance bacterial auto-aggregation in other LPS-deficient strains (34–36). LPS comprises three distinct regions: lipid A, a core polysaccharide, and an O-antigen (Fig. 3A). Silver-stained SDS-PAGE analysis of LPS profiles from wt and Δ*rfaH* strains revealed that the Δ*rfaH* strain lacks a single O-antigen unit of outer core LPS, consisting of lipid A, core, and one O-repeat unit, at both 26°C and 37°C (Fig. 3B). The lower molecular weight of the outer core in the Δ*rfaH* strain was previously observed in *Y. pseudotuberculosis* IP32953 and *Y. enterocolitica* and was associated with the absence of the single O-antigen unit (28, 37). Further investigation into the composition of lipid A species in both wt and Δ*rfaH* strains using matrix-assisted laser desorption/ionization – time of flight (MALDI-TOF) analysis revealed no differences between the two strains at either temperature (Fig. 3C and D). This suggests that the lower molecular weight of the LPS produced by the Δ*rfaH* strain may result from insufficient O-antigen biosynthesis, as the operon responsible for O-antigen biosynthesis, which is known to be regulated by RfaH, has an *ops* sequence located in the upstream region of the operon (Fig. 3E).

To determine whether the Δ*rfaH* phenotypes are due to defective O-antigen biosynthesis, we constructed several mutant strains deficient in O-antigen biosynthesis. To do this, we deleted the gene encoding for CDP-6-deoxy-delta 3,4-glucoseen reductase (Δ*ddhD*), an enzyme essential for CDP-ascarylose production during the biosynthesis of O-antigens (38). Moreover, we deleted a sizable DNA region, including seven genes (*gmd*, *fcI*, *manC*, *wbyQ*, *gne*, *manB*, and *wzz*), toward the end of the O-antigen operon to severely attenuate O-antigen biosynthesis (called Δ*O-aB*) (Fig. 3E). Additionally, we generated a mutant strain with a modified *ops* sequence located upstream of the O-antigen biosynthesis operon by introducing six nucleotide substitutions inside and in the flanking regions of the *ops* sequence (called *m-ops*) (Fig. 3E). LPS profiling showed that all three mutant strains exhibited a lower molecular weight of LPS outer core profiles similar to that of the Δ*rfaH* strain (Fig. 3F). The defective LPS profile of the *m-ops* strain provides evidence that RfaH positively regulates the O-antigen biosynthesis operon. Microscopy analysis of these O-antigen mutants under NaCl, BNG, and 37°C conditions revealed phenotypes mirroring those of the Δ*rfaH* strain. Following BNG treatment, these mutants exhibited similar aggregation phenotypes (Fig. 3G). Thus, the aggregation observed in the Δ*rfaH* strain is likely due to indirect effects from defective O-antigen biosynthesis.

### The global effect of RfaH on the *Y. pseudotuberculosis* transcriptome

**Fig 4:**
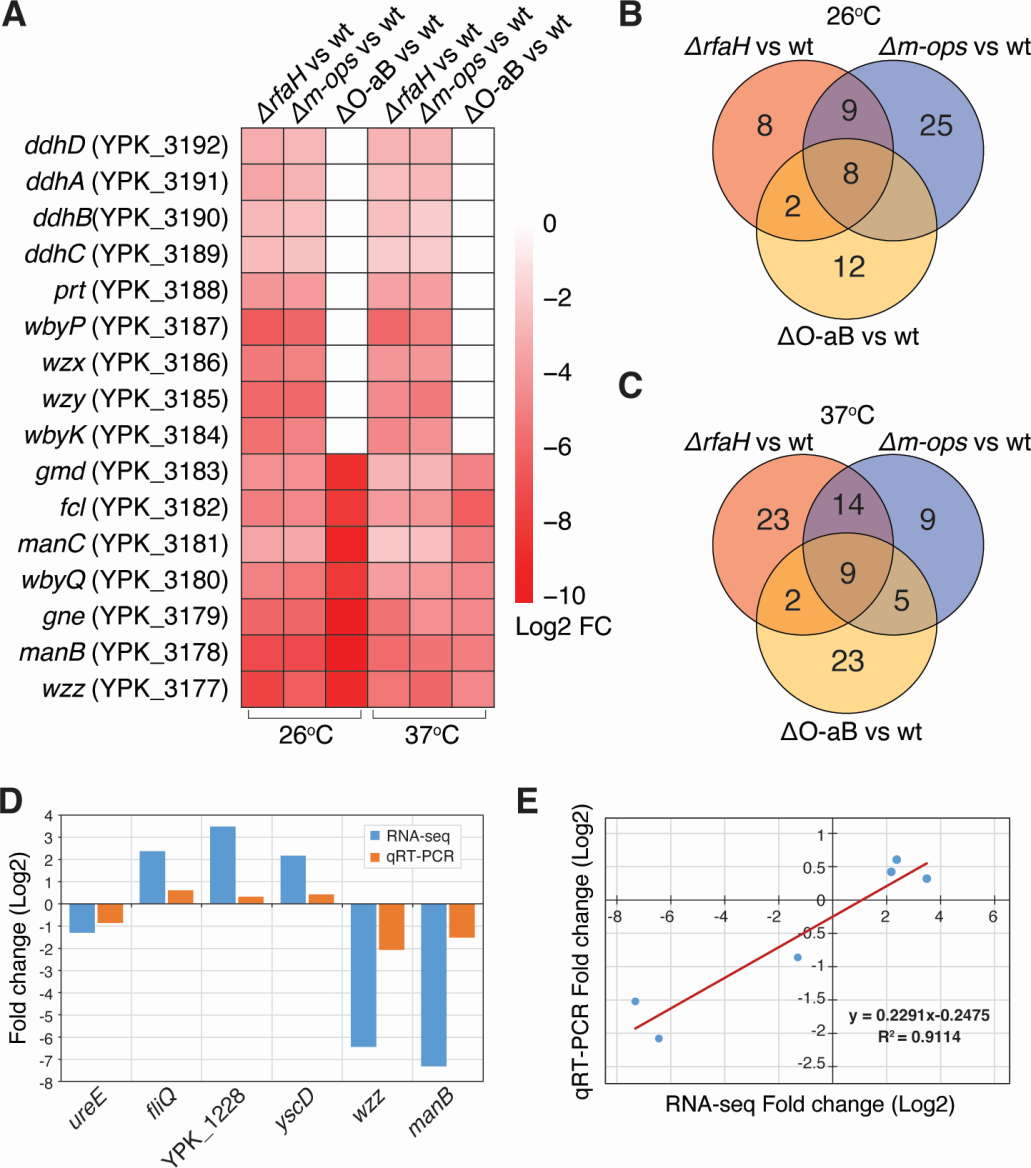
Differential gene expression profile of Δ*rfaH*, *m-ops*, Δ*O-aB* mutants, and wild-type strains with validation of the gene expression results by quantitative real-time PCR (qRT-PCR). (**A**) Heatmap highlighting low expression profiles of O-antigen biosynthesis operon genes in Δ*rfaH*, *m-ops*, and Δ*O-aB* versus the wt strain at 26°C and 37°C. (**B and C**) Venn diagrams highlighting differential gene expression patterns in Δ*rfaH*, *m-ops*, and Δ*O-aB* versus the wt strain at (**B**) 26°C and (**C**) 37°C. (**D**) Bar plot comparing RNA-seq and qRT-PCR expression levels of six randomly selected genes. The *x*-axis represents the genes, and the *y*-axis represents the log_2_ fold change from both RNA-seq and qRT-PCR. (**E**) Linear regression analysis of expression level between RNA-seq and qRT-PCR data. The *x*-axis represents the log_2_ fold change of RNA-seq, and the *y*-axis indicates the log_2_ fold change of qRT-PCR.

To investigate other potential genes or operons regulated by RfaH, we performed RNA-seq on Δ*rfaH* and wt strains grown at 26°C and 37°C. To distinguish genes whose expression regulation is affected specifically by defective O-antigen biosynthesis in the absence of RfaH, we included *m-ops* and Δ*O-aB* strains, both of which have defective O-antigen synthesis. Differential expression analysis revealed consistent and significant downregulation of all genes in the O-antigen biosynthesis operon (e.g., *manB, gne, fcl, gmd, wzx, wz*y) in both Δ*rfaH* and *m-ops* mutants compared to wt at both temperatures (Fig. 4A), consistent with the known role of RfaH in promoting efficient transcription elongation of long operons. In contrast, the Δ*O-aB* strain showed reduced expression only of the seven deleted genes (Fig. 4A) within the O-antigen operon, confirming the structural basis of the disruption.

Notably, analysis of uniquely differentially expressed genes (DEGs) revealed that Δ*rfaH* regulated a distinct set of genes not altered in the *m-ops* or Δ*O-aB* mutants. At 26°C, eight DEGs were exclusively regulated in Δ*rfaH* (Fig. 4B), including downregulated genes encoding a thiosulfate transporter (*cysP*), ribosomal protein (*rpmH*), a putative adhesin/hemolysin, and flagellar rod protein (*flgF*) (Table 1), suggesting additional roles for RfaH in motility, translation, and membrane physiology beyond O-antigen regulation. In contrast, genes encoding a putative lipoprotein (*ynbE*) and a virulence plasmid located transposase (piBX002) were upregulated, possibly due to the loss of RfaH-mediated transcriptional repression of stress-related genes or mobile elements (Table 1). At 37°C, the transcriptomic impact of Δ*rfaH* was even broader, with 23 Δ*rfaH*-specific DEGs (Fig. 4C). These included downregulated genes involved in membrane transport (e.g., holin, monosaccharide-transporting ATPase) and phage-related or tail-associated proteins, along with upregulated genes linked to phage activity (e.g., *alpA*), fimbriae, and type VI secretion system components. The increased number of Δ*rfaH*-specific DEGs at host temperature suggests additional regulatory roles for RfaH under infection-relevant conditions, potentially modulating envelope structure, mobility, and stress adaptation (Table 1). Notably, no *ops-*like motifs were detected in the regulatory part of any of these genes or operons.

**TABLE 1:** Log_2_ fold change of the DEGs regulated in each strain relative to wt at 26°C and 37°C^*a*^

Locus	Gene	Description	Log_2_ fold change (26°C)			Log_2_ fold change (37°C)		
			Δ*rfaH*	*m-ops*	Δ*O-Ab*	Δ*rfaH*	*m-ops*	Δ*O-Ab*
YPK_3937	*rfaH*	Transcriptional activator RfaH	−8.47	—	—	−6.68	—	—
YPK_0406		Putative cytoplasmic protein	−3.10	—	—	—	—	—
YPK_4249	*rpmH*	50S ribosomal protein L34	−1.74	—	—	—	—	—
YPK_0582		Putative adhesin/hemolysin	−1.14	—	—	—	—	—
YPK_2421	*flgF*	Flagellar basal body rod protein FlgF	−1.10	—	—	—	—	—
YPK_1408	*cysP*	Sulfate ABC transporter	−1.05	—	—	—	−1.03	—
YPK_1920	*ynbE*	Putative lipoprotein	1.09	—	—	—	—	—
piBX002		Transposase	1.96	—	—	—-	—	—
YPK_1388	*napB*	Nitrate reductase cytochrome c-type subunit	—	−1.64	—	—	—	—
YPK_0379		Glycosidase	—	−1.33	—	—	—	—
YPK_1134	*ureE*	Urease accessory protein UreE	—	−1.30	—	—	—	—
YPK_1133	*ureC*	Urease subunit alpha, UreC	—	−1.28	—	—	—	—
YPK_1137	*ureD*	Urease accessory protein UreD	—	−1.13	—	—	—	—
YPK_1135	*ureF*	Urease accessory protein UreF	—	−1.09	—	—	—	—
YPK_1136	*ureG*	Urease accessory protein UreG	—	−1.04	—	—	—	—
YPK_3760	*nlp*	Putative transcriptional regulator Nlp	—	−1.28	—	—	—	—
YPK_4113	*glpK*	Glycerol kinase	—	−1.04	—	—	—	—
YPK_RS05760		Hypothetical protein	—	−1.06	—	—	—	—
YPK_3632		Hypothetical protein	—	−1.04	—	—	—	—
piBX084		Lipoprotein Virg	-—	1.03	—	—	—	—
YPK_3390	*cybB*	Cytochrome b561	—	1.03	—	—	—	—
YPK_3948	*cstA*	Carbon starvation protein CstA	—	1.07	—	—	—	—
YPK_0725	*cadC*	Putative transcriptional regulator CadC	—	1.19	—	—	—	—
YPK_0617		AntA/AntB antirepressor domain-containing protein	—	1.33	—	—	—	—
YPK_1316		Insertion element protein	—	4.02	—	—	—	—
YPK_1642		Hypothetical protein	—	1.10	—	—	—	—
YPK_1153		Hypothetical protein	—	1.14	—	—	—	—
piBX059		Hypothetical protein	—	1.24	—	—	—	—
YPK_2209		Hypothetical protein	—	1.28	—	—	—	—
YPK_3090		Hypothetical protein	—	3.68	—	—	—	—
YPK_1734		Hypothetical protein	—	1.39	—	—	—	—
YPK_2076		Hypothetical protein	—	2.41	—	—	—	—
YPK_0840		Hypothetical protein	—	3.48	—	—	—	—
YPK_0192		Hypothetical protein	—	—	−3.64	3.58	4.50	3.56
YPK_RS05880		Hypothetical protein	—	—	−3.52	—	—	—
YPK_RS07695		Hypothetical protein	—	—	−2.46	—	—	—
YPK_0193		Hypothetical protein	—	—	−1.56	—	—	—
YPK_0848		Transposase IS200 family protein	—	—	−1.23	1.40	1.07	—
YPK_4237		Extracellular solute-binding protein	—	—	−1.04	—	—	—
YPK_1972		Hypothetical protein	—	—	1.20	—	—	—
YPK_3963		Hypothetical protein	—	—	1.54	—	—	—
YPK_RS20560		Hypothetical protein	—	—	1.95	—	—	—
YPK_1458		Hypothetical protein	—	—	2.38	—	—	—
YPK_RS05195		Hypothetical protein	—	—	2.46	—	—	—
YPK_2234		Transposase IS200-family protein	—	—	4.95	—	—	—
YPK_RS11665		Holin	—	—	—	−3.40	—	—
YPK_2349		Tail E family protein	—	—	—	−3.06	—	—
YPK_3401		Monosaccharide-transporting ATPase	—	—	—	−1.16	—	—
YPK_2845		Hypothetical protein	—	—	—	−4.23	—	—
YPK_0384		Hypothetical protein	—	—	—	−4.10	—	—
YPK_2886		Hypothetical protein	—	—	—	−3.02	—	—
YPK_0760		Hypothetical protein	—	—	—	−2.01	—	—
YPK_2479		Hypothetical protein	—	—	—	−1.41	—	—
YPK_2211		Hypothetical protein	—	—	—	−1.23	—	—
YPK_4042	*fimA-5*	Fimbrial protein	—	—	—	1.46	—	—
YPK_3140	*alpA*	Phage transcriptional regulator AlpA	—	—	—	1.18	—	—
YPK_3284		Type 11 methyltransferase	—	—	—	1.00	—	—
YPK_3565		Type VI secretion protein	—	—	—	1.28	—	—
piBX060		Uncharacterized protein pYV0051	—	—	—	1.60	—	—
YPK_1029		Hypothetical protein	—	—	—	1.01	—	—
YPK_1785		Hypothetical protein	—	—	—	1.12	—	—
YPK_0362		Hypothetical protein	—	—	—	1.22	—	—
YPK_1433		Hypothetical protein	—	—	—	1.40	—	—
YPK_RS18305		Hypothetical protein	—	—	—	1.00	—	—
YPK_2893		Hypothetical protein	—	—	—	3.34	—	—
YPK_0213		Hypothetical protein	—	—	—	3.66	—	—
YPK_0817		Hypothetical protein	—	—	—	4.50	—	—
YPK_0342	*thiH*	Thiamine biosynthesis protein ThiH	—	—	—	—	−1.01	—
YPK_1610		Alcohol dehydrogenase zinc-binding	—	—	—	—	−1.02	—
YPK_2130		Spermidine export protein Mdt	-—	—	—	—	−1.50	—
YPK_RS09520		Hypothetical protein	—	—	—	—	1.70	—
YPK_2612		Hypothetical protein	—	—	—	—	1.76	—
YPK_0953		Hypothetical protein	—	—	—	—	1.81	—
YPK_0705	*fliQ*	Flagellar biosynthetic protein FliQ	—	—	—	—	2.37	—
YPK_1228		Hypothetical protein	—	—	—	—	3.48	—
YPK_2533		Transposase IS200 family protein	—	—	—	—	—	−5.66
YPK_0940		Small terminase subunit	—	—	—	—	—	−3.61
YPK_2424	*flgC*	Flagellar basal body rod protein FlgC	—	—	—	—	—	−2.45
YPK_0477		Putative phage-like protein	—	—	—	—	—	−2.38
YPK_2395	*fliJ*	Flagellar export protein FliJ	—	—	—	—	—	−2.05
YPK_2400	*fliN*	Flagellar motor switch protein FliN	—	—	—	—	—	−1.08
YPK_3933	*fadB*	Fatty oxidation complex, alpha subunit FadB	—	—	—	—	—	−1.00
YPK_1605	*rhtA*	Inner membrane transporter RhtA	—	—	—	—	—	−1.37
YPK_0727		Hypothetical protein	—	—	——		—	−3.37
YPK_1149		Hypothetical protein	—	—	—	—	—	−2.07
YPK_3058		Hypothetical protein	—	—	—	—	—	−2.06
YPK_3414		Hypothetical protein	—	—	—	—	—	−1.44
YPK_1383		Hypothetical protein	—	—	—	—	—	−1.25
YPK_0121	*uspB*	Universal stress protein UspB	—	—	—	—	—	1.05
YPK_3991		sn-glycerol-3-phosphate dehydrogenase subunit A	—	—	—	—	—	1.05
YPK_3201		Hypothetical protein	—	—	—	—	—	1.09
piBX027		Insertion element ISR1	—	—	—	—	—	1.11
YPK_RS17330		Hypothetical protein	—	—	—	—	—	1.13
YPK_3210		50S ribosomal protein L31	—	—	—	—	—	1.13
YPK_1385	*napF*	Ferredoxin-type protein NapF	—	—	—	—	—	1.15
YPK_1143	*ccrB-2*	CrcB protein	—	—	—	—	—	1.49
YPK_2133		Hypothetical protein	—	—	—	—	—	2.06
YPK_3916	*yscD*	YscD/HrpQ, type III secretion apparatus protein	—	—	—	—	—	2.17
YPK_3177^†^	*wzz*	Ferric enterobactin transport protein FepE	−7.60	−6.44	−9.03	−5.39	−6.15	−4.73
YPK_3178^†^	*manB*	Phosphomannomutase	−7.25	−7.32	−10.05	−5.85	−5.55	−5.06
YPK_3179^†^	*gne*	NAD-dependent epimerase/dehydratase	−6.09	−6.15	−10.21	−5.48	−4.37	−4.65
YPK_3180^†^	*wbyQ*	Glycosyl transferase family 2 protein	−5.04	−5.44	−8.23	−3.77	−3.97	−4.70
YPK_3181^†^	*manC*	Mannose-6-phosphate isomerase	−3.44	−3.44	−9.60	−2.22	−2.45	−5.14
YPK_3182^†^	*fcl*	NAD-dependent epimerase/dehydratase	−5.16	−4.74	−8.28	−3.93	−4.22	−6.43
YPK_3183^†^	*gmd*	GDP-mannose 4,6-dehydratase	−4.38	−4.35	−8.78	−2.87	−3.01	−4.92
YPK_3184^†^	*wbyK*	Mannosyltransferase	−5.76	−4.95	—	−4.81	−4.34	—
YPK_3185^†^	*wzy*	O-antigen biosynthesis protein Wxy	−5.99	−6.05	—	−4.58	−5.19	—
YPK_3186^†^	*wzx*	LPS side chain defect: putative O-antigen transferase	−5.18	−5.05	—	−4.07	−4.09	—
YPK_3187^†^	*wbyP*	Glycosyl transferase family 2	−6.61	−6.09	—-	−6.02	−5.05	—
YPK_3188^†^	*prt*	NAD-dependent epimerase/dehydratase	−4.14	−3.86	—	−3.66	−3.77	—
YPK_3189^†^	*ddhC*	CDP-4-dehydro-6-deoxyglucose reductase	−2.69	−2.38	—	−1.95	−2.04	—
YPK_3190^†^	*ddhB*	CDP-glucose 4,6-dehydratase	−2.81	−2.53	—	−2.43	−1.97	—
YPK_3191^†^	*ddhA*	Glucose-1-phosphate cytidylyltransferase	−3.49	−2.96	—	−2.59	−2.73	—
YPK_3192^†^	*ddhD*	Oxidoreductase FAD/NAD(P)-binding domain protein	−3.19	−2.81	—	−3.03	−2.94	—
YPK_0866		Hypothetical protein	—	—	—	—	−1.88	−1.09
YPK_0280	*bfd*	Bacterioferritin-associated ferredoxin	—	—	—	—	−1.39	−1.20
YPK_1160		Hypothetical protein	—	—	—	—	1.01	1.19
YPK_1837	*arnF*	Undecaprenyl phosphate-alpha-L-ara4N flippase	—	—	—	—	1.24	1.21
YPK_3798		Hypothetical protein	—	—	—	—	4.62	3.67
YPK_0142		Hypothetical protein	—	—	—	1.01	1.01	—
YPK_1770		Hypothetical protein	—	—	—	1.19	1.35	——
YPK_1304		Type VI secretion system effector, Hcp1 family	—	—	—	1.72	1.23	—
YPK_3926		Transposase IS3/IS911 family protein	—	—	—	−22.84	—	−22.88
YPK_1861		Hypothetical protein	—	—	—	−3.19	-	−4.10
YPK_1389	*napC*	Cytochrome c-type protein NapC	−1.44	−1.35	−1.51	—	—	—
YPK_1246		Hypothetical protein	1.01	—	1.29	—	—	—
YPK_3328		Polar amino acid ABC transporter	3.33	—	3.39	3.51	3.22	—
YPK_0631		Hypothetical protein	—	—	—	1.06	1.05	1.04

^*a*^ — indicates failure to meet the significance cutoff or undetected. † indicates DEGs whose promoter regions contain the *ops* sequence.

For the *m-ops* mutant, 25 DEGs were exclusively regulated at 26°C, including strong downregulation of the urease operon (*ureC, ureD, ureE, ureF, ureG*), which remained unaffected in both Δ*rfaH* and Δ*O-aB* mutants (Table 1). These findings suggest that the 5′ untranslated region of the O-antigen biosynthesis operon, including the *ops* sequence, a known RfaH recruitment signal, may influence additional regulatory pathways beyond O-antigen biosynthesis. In the Δ*O-aB* strain, 35 exclusive DEGs (Table 1) were identified across both temperatures, consistent with the complete removal of key O-antigen biosynthetic genes. These changes likely reflect secondary responses to the total loss of LPS side chain structure, potentially involving envelope stress, metabolic shifts, or activation of mobile genetic elements. Eight DEGs were shared by all three mutants and corresponded to core O-antigen biosynthesis genes, all consistently downregulated relative to wt, highlighting their dependency on both structural and transcriptional integrity of the operon.

To validate the RNA-seq findings, we performed quantitative real-time PCR (qRT-PCR) on six representative genes. Expression patterns were consistent between qRT-PCR and RNA-seq data, with a strong correlation (*R*² = 0.8807), thereby confirming the robustness of our transcriptomic analysis (Fig. 4D and E).

### RfaH is required for full *in vivo* virulence of *Y. pseudotuberculosis*

To assess the role of RfaH in *Y. pseudotuberculosis* virulence in a mouse model, we performed low-dose oral infection in FVB/N mice using the Δ*rfaH* mutant and wt strains. Thereafter, we followed *Y. pseudotuberculosis* colonization with the presence or absence of a bioluminescent signal produced by a bioluminescent reporter system introduced in the virulence plasmid and monitored with *In Vivo* Imaging System (IVIS).

**Fig 5:**
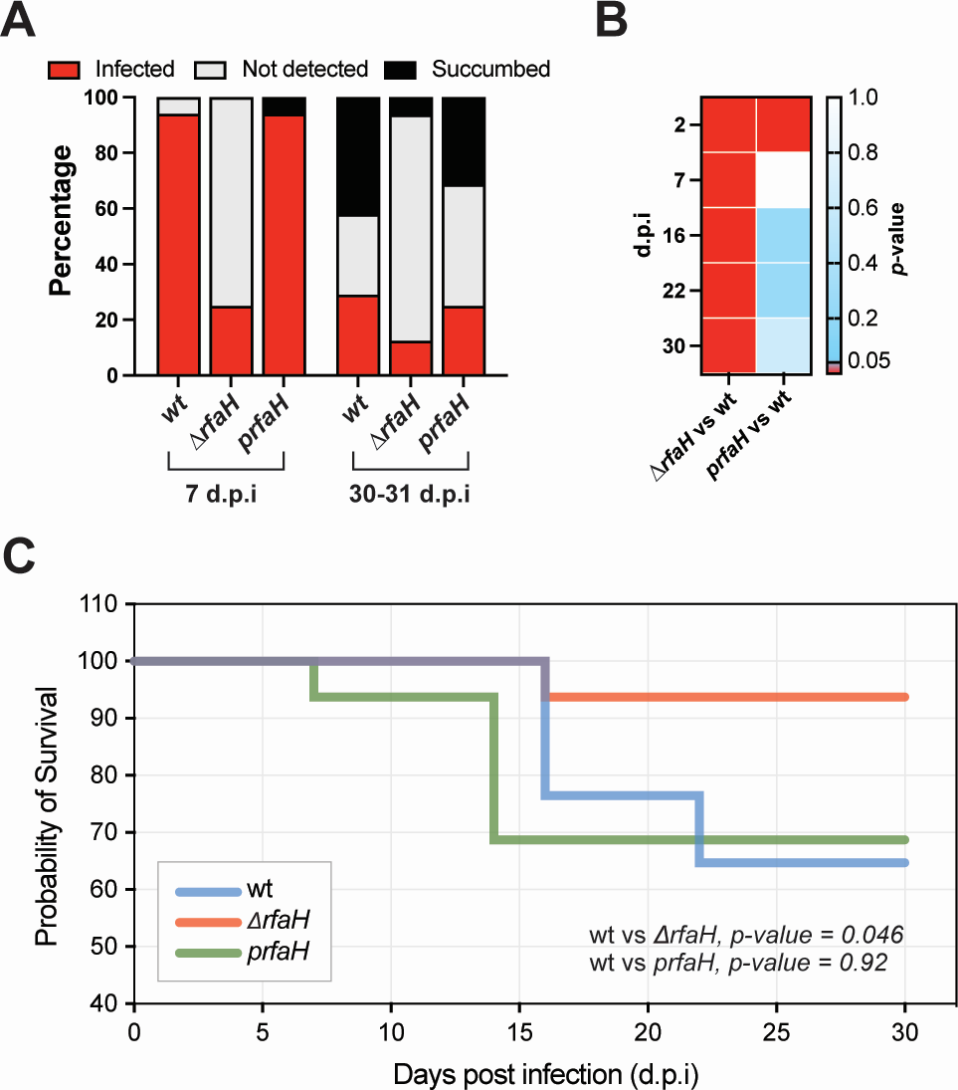
Loss of RfaH attenuates virulence in low-dose oral infection of FVB/N mice and can be complemented by *trans* expression of *rfaH*. (**A**) Infection profile of FVB/N mice infected with a low dose of wt (*n* = 17), Δ*rfaH* (*n* = 16), and *prfaH* (*n* = 16) *Y. pseudotuberculosis* at early (7 d.p.i.) and persistent (30–31 d.p.i.) infection phases. Mice were orally infected with overnight bacterial cultures, and the infection process was monitored daily post-infection for wt, Δ*rfaH* mutant, and *prfaH* strains. (**B**) Heatmap showing differences (by *P*-values) in the profiles of mice that were infected, not detected, or succumbed, comparing wt versus Δ*rfaH* and *prfaH* strains separately at different time points. *P*-values were calculated using Fisher’s exact test. (**C**) Survival rates of mice infected with wt, Δ*rfaH*, and *prfaH* strains. Statistical significance was determined using the log-rank (Mantel-Cox) test.

While up to 94% of the mice were infected with the wt strain, only 19% of the mice were infected with the Δ*rfaH* mutant strain (Fig. S1A). Furthermore, 36% of wt-infected mice developed acute infection and succumbed to the infection by 16 days post-infection (d.p.i.), whereas none of the Δ*rfaH*-infected mice showed symptoms of acute infection (Fig. S1A). Interestingly, one Δ*rfaH*-infected mouse developed symptoms of acute infection, despite the absence of or undetectable bioluminescent signal. While this suggests the possibility of transient colonization or IVIS sensitivity limitations, we cannot rule out unrelated causes of illness in this individual animal. Moreover, the ability of the Δ*rfaH* strain to establish persistent infection was reduced, with only two mice maintaining infection, compared to five mice infected with the wild-type strain up to 30 d.p.i. (Fig. S1A). This reduction is likely influenced by the requirement for RfaH during the initial stages of infection, with a notable number of mice remaining uninfected. Notably, the attenuation observed in the Δ*rfaH* mutant in the mouse oral infection model could be complemented with the expression of *rfaH* in *trans* (Fig. S1A). Infection with the *prfaH* complementation strain resulted in four mice developing persistent infection, indicating that RfaH is required to establish persistent infection in mice. Additionally, we monitored the presence of *prfaH* during infection by continually screening for antibiotic resistance, encoded on the plasmid that encodes *rfaH*, on *Y. pseudotuberculosis* that was shed through feces (Fig. S1B). Our results confirmed that *prfaH* was still present even after 31 d.p.i., and with a high bacterial load after 10 d.p.i. (Fig. S1B). Overall, the infection profile of Δ*rfaH*-infected mice showed attenuation in virulence capacity at both the early (7 d.p.i.) and persistent (30–31 d.p.i.) phases, whereas this attenuation was complemented in mice infected with the *prfaH* strain (Fig. 5A). Moreover, these differences were statistically significant at all monitored time points (Fig. 5B). Further analysis of the survival of mice infected with different strains showed significantly higher survival of mice infected with Δ*rfaH* compared to mice infected with strains expressing this regulator (Fig. 5C).

## DISCUSSION

In the present study, we found that the RfaH expression reaches the highest level at the stationary phase under various infection-related *in vitro* conditions. This correlates with the observed upregulation of RfaH during persistent infection, where the transcriptome of persistently colonized *Y. pseudotuberculosis* is more similar to bacteria in the stationary phase than to that in growing bacteria (11). Similarly, Rojas et al. (30) reported that the expression of RfaH increased up to twofold when the growth of *Salmonella* reached the late exponential phase and remained high throughout the stationary phase. Moreover, we also observed RfaH upregulation under multiple stress conditions, including NaCl, bile salts, low pH, oxidative stress, temperature, and BNG, implying a possible role of RfaH in stress adaptation and survival in harsh conditions. However, the *rfaH-lacZ* translational fusion utilized in this study reports regulation up to translation initiation but does not account for potential post-initiation effects such as differential protein stability or regulation during elongation.

Another signature of bacteria in the persistent infection stage was the upregulation of genes involved in envelope perturbation (11). In this study, LPS profiling of strains mutated in different RfaH-dependent features highlighted uniformly defective LPS profiles, similar to the Δ*rfaH* strain. These observations are consistent with RfaH acting as a positive regulator of the O-antigen biosynthesis operon. Defective LPS biosynthesis impacted multiple bacterial surface processes, including clumping phenotypes of the various mutant strains, which were comparable to the Δ*rfaH* strains. In *Salmonella*, RfaH-regulated LPS has been reported to play a key role in the interaction between cells and the environment, contributing to virulence (30). Similarly, regulation of LPS has been reported as a common strategy for bacteria to change their surface and adapt to new environments, such as increased temperature and exposure to different chemicals (39). These results suggest that LPS-defective bacteria will have challenges adapting during infection. Previous studies in *E. coli* have shown that intestinal colonization was impacted by the regulatory action of RfaH on LPS-core production (40). Surprisingly, the LPS defect might also be responsible for the aggregation phenotype of Δ*rfaH,* as shown in this study. The reason for this is unclear, but it might be clustering *per se* or that the LPS defect affects the organization of membrane components in a way that hinders flagella function or the reduced expression of the *flgF* gene, which encodes the basal body rod protein that transmits motor torque from the hook to the filament, facilitating bacterial movement (41). In *Aeromonas hydrophila*, Qin et al. (42) reported that FlgF substituted FlgC in the formation of bacterial flagellar rod while preserving the presence of flagella. However, disruption of the *flgC* led to a 42% reduction of bacterial motility, chemotaxis, and adhesion (42). Consistent with these reports, downregulation of *flgF* in the Δ*rfaH* mutant at 26°C may contribute to reduced motility or adhesion, even though flagella are still present (Fig. 2C). Previous studies have reported that deletion of the *rfaH* gene attenuates bacterial virulence in several ways, including reduction of capsules and intact LPS, hemin receptor, and alpha-hemolysin during infection (29). The *E. coli* A192PP Δ*rfaH* mutant was highly susceptible, with no colonies detected after 30 min of incubation in 22% normal human serum or heat-inactivated serum (56°C for 30 min) (43). In this study, our transcriptome analysis highlighted that deletion of *rfaH* exclusively affected the expression of a specific set of genes at 26°C and 37°C, compared to the wt strain (Table 1). These genes might shed light on the global regulatory function of RfaH in *Y. pseudotuberculosis*. Genes encoding cytoplasmic protein, *flgF*, and hemolysin were downregulated in the Δ*rfaH* strain compared to the wt strain at 26°C. Similarly, genes encoding holin and monosaccharide-transporting ATPase were downregulated at 37°C (Table 1). In *E. coli*, the alpha-hemolysin expression has been reported to be under the control of RfaH (44). A study on *Y. enterocolitica* reported a decrease in the amount of Mg^2+^ transport ATPase proteins and phosphate ABC transporter in the YeO3-Δ*rfaH* strain (37). As suggested by Nagy et al. (23), this might be an adjustment in response to decreased LPS production due to the RfaH deletion, resulting in reduced demand for sugars and energy. In the *Y. pseudotuberculosis* Δ*rfaH* strain, the expression of genes encoding lipoprotein and transposase was enhanced at 26°C, while those encoding type VI secretion protein, transcriptional regulator AlpA, type 11 methyltransferase, and fimbrial protein were upregulated at 37°C (Table 1). A previous study of *Acinetobacter baumannii* reported that an LPS-deficient strain displayed increased expression of genes encoding cell envelope and membrane biogenesis, like lipoproteins and lipoprotein transport system (45). It is therefore possible that alteration of the lipoprotein content of the outer membrane is a response to LPS loss. Fimbrial proteins and type II methyltransferases have been reported to play key roles in the virulence and defense of several bacteria, including *E. coli* (46), *Salmonella* (47), *Avibacterium paragallinarum* (48), *Metamycoplasma hominis* (49), and *Xanthomonas axonopodis* pv. *Glycines* (50). Similarly, an AlpA positively regulated a programmed cell death pathway linked to the virulence of *Pseudomonas aeruginosa* (51), while the type VI secretion system has been identified within pathogenicity islands of *Salmonella* (52), as well as in the survival of *Vibrio anguillarum* and *V. cholerae* under stress conditions (53, 54). Interestingly, the high number of hypothetical genes among those regulated by RfaH suggests that much remains unknown about this transcriptional regulator. However, further analysis of these genes could provide valuable insights into potential mechanisms of *rfaH* regulation in infection. Notably, the stronger transcriptional impact observed at 37°C suggests that RfaH plays a more extensive regulatory role under host-like conditions. In contrast, gene expression changes at 26°C likely reflect environmental adaptation or priming for host colonization, consistent with the observed similarity between 26°C-grown bacteria and the transcriptome of persistent-phase *Y. pseudotuberculosis*.

The loss of RfaH severely attenuated the virulence of *Y. pseudotuberculosis* upon infection, as only two mice infected with the Δ*rfaH* strain could keep the infection up to 30 d.p.i. as compared to five mice infected with the wt strain (Fig. S1A). These highlight the significant role of RfaH in both the initial phase of infection and in the adaptation and maintenance of the pathogen at later stages. Notably, O-antigen-defective strains of *Y. pseudotuberculosis* and *Y. enterocolitica* showed attenuation in both mouse and rabbit infection models (55–57), indicating that an alteration in the cell surface might contribute to the attenuation observed in the *rfaH* deletion mutant. Similarly, the inactivation of RfaH dramatically decreased the virulence of the uropathogenic *E. coli* strain (29). However, a previous study reported that deleting the *rfaH* gene in *Y. pseudotuberculosis* did not reduce its virulence in BALB/c mice during acute infection (28). However, that study used a different *Y. pseudotuberculosis* strain (IP32953) and a different mouse model (BALB/c) for infection. We have previously demonstrated that FVB/N mice are more suitable for the establishment of persistent infection by the YPIII strain than BALB/c mice. One difference between the mouse strains used in the two studies is their major histocompatibility complex class I haplotype: BALB/c mice possess H-2^d^, while FVB/N mice have H-2^q^. The susceptibility of FVB/N mice to persistent murine encephalomyelitis virus infection and the increased resistance conferred by an H-2^b^ transgene highlight the significant role of major histocompatibility complex (MHC) haplotype variation in determining host susceptibility to infectious agents (58). This suggests that different outcomes of *rfaH* deletions could simply be due to different mouse models used in different infections. Moreover, although *Y. pseudotuberculosis* YPIII and IP32953 strains have the same O-antigen biosynthesis operon structure and upstream *ops* sequence, IP32953 is known to be more virulent and exhibit greater colonization capacity in mice than YPIII (59). This suggests that strain-specific factors, beyond *rfaH* and O-antigen production, significantly influence virulence in mouse models of *Y. pseudotuberculosis* infection.

Mutations in the *ops* region can impair RfaH binding, leading to premature transcription termination or improper transcription of downstream genes, thereby reducing their expression. Interestingly, our RNA-seq analysis revealed that the *ops* mutation affected the transcriptional regulation of several genes that are not regulated by RfaH. This finding underscores a possible distinct regulatory role for this *ops* element, independent of RfaH, in the expression of specific genes. Notably, analysis of read mappings of the O-antigen biosynthesis operon’s 5′ untranslated region, surrounding the *ops* region, revealed a unique mapping profile in the *m-ops* strain. The relatively low coverage of the GGGGGG sequence downstream of the *ops* sequence in the wt and Δ*rfaH* strains, compared to the flanking regions, was absent in the *m-ops* strain (Fig. S2). This observation suggests the presence of a potential RNA motif that may have been disrupted by a single nucleotide substitution in the *m-ops* strain, possibly indicating the action of an RNase that processes the 5′ UTR of the O-antigen biosynthesis operon. Consequently, a possible cleavage of this region may result in an approximately 136 nt long 5′ UTR, which may function as a regulatory non-coding RNA precisely modulating the expression of genes uniquely regulated in the *m-ops* strain. In *E. coli*, the *ops* sequence leads to the formation of a DNA hairpin in the non-template strand (60). This hairpin is critical for RfaH recruitment, thereby stabilizing the paused complex and enabling subsequent transcriptional activation (61). Mutations within the *ops* sequence, like in our *m-ops* strain, might not only disrupt RfaH binding but also destabilize the formation of this hairpin, thereby abolishing RNAP pausing. Thus, the low read coverage downstream of *ops* sequence in the *m-ops* strain can be alternatively explained by the loss of this structured pause element, rather than (or in addition to) the action of a hypothetical non-coding RNA. However, all these hypotheses are beyond the scope of the current study, and they require further investigation to elucidate the novel regulatory role of the 5′ UTR in the O-antigen biosynthesis operon.

## MATERIALS AND METHODS

### Strains and growth conditions

The wt strain in this study was the kanamycin-resistant bioluminescent *Y. pseudotuberculosis* YPIII/pIBX strain (Table S1). The YPIII strain is a well-studied model for *Y. pseudotuberculosis* pathogenesis (10, 11). The strains were cultured overnight at 26°C and 220 rpm in Luria broth (LB) supplemented with 50 µg/mL kanamycin. The samples were co-cultured in the morning to a starting OD of 0.05 and grown in LB at 26°C until the exponential phase before being aliquoted for several *in vitro* growth and total RNA purification experiments at 26 and 37°C. For stress conditions, the LB was supplemented with 125 mM NaCl, 0.5% bile, 0.2% glucose, and a combination of all three (LB-BNG).

### Mutant and other strain constructions

To generate an in-frame deletion of a single gene or large genomic region of DNA, a 200-nucleotide flanking region in both ends of the region of interest was PCR amplified and ligated together with *Sal*I and *Bgl*I (New England Biolabs, Inc.) into pDM4 (62) using the In-Fusion HD Cloning Kit (Clontech Laboratories, Inc.) according to the manufacturer’s instructions. The same strategy was employed for multiple nucleotide substitutions by using degenerate PCR primers at the PCR amplification step. The plasmids were transformed into *E. coli* DH5αλpir, and positive clones were selected on chloramphenicol (50 µg/mL) containing agar plates, confirmed by colony PCR. Confirmed plasmid constructs were transformed into *E. coli* S17-1λpir conjugation strain for conjugal mating with *Y. pseudotuberculosis* YPIII/pIBX. Positive allelic exchange was selected as described previously (62). The mutant strains of Δ*rfaH*, *m-ops*, and Δ*O-aB* were finally confirmed with sequencing (Table S1). For the complementation of *rfaH*, the gene and its promoter sequence were cloned into the *BamH*I and *HindIII* sites of pMMB66HE, resulting in *prfaH*. The promoter region of *rfaH* and a part of the downstream region encoding the first 13 amino acids were fused to promoterless *lacZ* in pFU61, where ColE1 had been exchanged with the low-copy replicon pSC101.

### Mouse infection and bioluminescent imaging

Bacteria were subcultured on LB agar plates supplemented with kanamycin (50 µg/mL). For infection, the bacteria were grown overnight in LB at 26°C, and concentrations were estimated by absorbance at OD_600_. Cultures were resuspended to 10^7^ colony forming units (CFUs)/mL in sterilized tap water supplemented with 150 mM NaCl. Viable count and drinking volume determined the infection dose. Eight-week-old female FVB/N (Taconic Farms, Inc.) mice were deprived of food and water for 16 hours before oral infection with ~10^7^ CFUs of wt *Y. pseudotuberculosis* YPIII (pIBX, harboring the luxCDBAE operon in the virulence plasmid pYV) or the corresponding Δ*rfaH* mutant strain, which were supplied in their drinking water for 6 hours. Mice were inspected frequently for signs of infection and to ensure that infected mice showing prominent clinical signs were euthanized promptly to prevent suffering. The infections were monitored up to 31 d.p.i. by an *in vivo* imaging system, IVIS Spectrum, which was based on detecting the light produced by luciferase activity encoded on the *luxCDBAE* operon (63). Before imaging, the mice were anesthetized using the XGI-8 gas anesthesia system (Caliper LifeSciences, Inc.), which allowed control throughout anesthesia. The oxygen mixed with 2.5% IsoFloVet (Orion Pharma, Abbott Laboratories Ltd., Great Britain) was used for the initial anesthesia, and 0.5% isoflurane in oxygen was used during imaging.

### β-Galactosidase assays

The *in vivo* transcriptome profile of persistent *Y. pseudotuberculosis* has been reported to resemble that of stationary-phase bacteria (11). Thus, we created a reporter vector with the *rfaH* promoter region, ribosome binding site, and first 13 amino acids fused to the *lacZ* coding sequence gene to screen for induction of RfaH expression by measuring β-galactosidase activity. The reporter vector was expressed in *trans* in wt *Y. pseudotuberculosis,* and the β-galactosidase activity was measured during growth in LB at 26°C. The expression level of RfaH was calculated by measuring the β-galactosidase activity of the strain with the RfaH-LacZ fusion. The β-galactosidase activity in this study was measured according to the method described by Zhang et al. (64). β-Galactosidase activity samples were taken every hour for the growth phase-dependent period, and absorbance at OD_600_ was recorded. On the other hand, we measured the RfaH activity after adding 125 mM NaCl, 0.5% bile salts, low pH with adding acetic acid, H_2_O_2_, temperature (37°C), and a combination of bile, NaCl, and glucose (BNG). The treatments were performed on bacterial cultures in LB at exponential (OD_600_: 0.4–0.5) and stationary (OD_600_: 2.2–2.4) phases and lasted for 1 hour, whereafter the activity of β-galactosidase was measured. All the experiments were repeated with six biological replicates, and enzyme activities (Miller units) were normalized according to cell density (OD_600_).

### Phenotypic analysis

The wt and Δ*rfaH Y. pseudotuberculosis* strains were grown as described in the strain and growth conditions section. To visualize bacterial morphologies, the bacterial samples were mounted on slides and visualized under a phase contrast microscope after 2 hours of exposure to stress conditions and when the bacterial culture reached OD_600_ = 0.8.

### Motility assay

Bacteria from overnight cultures were inoculated into LB and grown to OD_600_ = 0.8. Subsequently, they were treated with and without NaCl or BNG at 26°C for 2 hours. A 5 µL aliquot of each culture was spotted on LB with 0.25% agar. Plates were incubated at 26°C under aerobic conditions for 48 hours. The images of bacteria on the plates were monitored using a ChemiDoc XRS System (Bio-Rad).

### Visualization of flagella by atomic force microscopy

The wt and Δ*rfaH* of *Y. pseudotuberculosis* YPIII-pIBX strains were grown overnight, diluted 25 times with LB media in the morning, and then cultured for 2 hours at 26°C to an OD_600_ = 0.8. Immediately after the bacterial cultures reached the desired OD_600_ value, the growth medium was supplemented with 125 mM NaCl or 0.5% bile, 125 mM NaCl, and 0.2% glucose. Following a 2-hour treatment, the samples were centrifuged for 4 min at 1,500 rpm, washed once with 2 mM MgCl_2_, and resuspended in 50 μL–200 μL of the same solution. Ten microliters of each sample was placed on freshly cleaved ruby red mica (Goodfellow Cambridge Ltd., Cambridge), incubated for 5 min at room temperature, and blotted dry before being placed into a desiccator for at least 2 hours. Images were collected by a Nanoscope V atomic force microscope (Bruker software) using ScanAsyst in the air with ScanAsyst cantilevers at a scan rate of approximately 0.9–1 Hz. The resulting images were flattened and/or plane-fitted in both axes using Bruker software and presented in amplitude (error) mode.

### LPS analysis

Diluted overnight cultures were grown at 26°C until OD_600_ = 0.8. Thereafter, 1 mL cultures were centrifuged, and pellets were resuspended in 100 µL lysis buffer (100 mM Tris-HCl, pH 6.8, 1.5% SDS, 1.5% β-mercaptoethanol) and boiled for 10 min. After that, samples were subjected to an overnight treatment with 80 µg proteinase K, followed by SDS-PAGE gel electrophoresis. The gels were stained with Pierce Silver Staining Kit (Life Technologies, Inc.) according to the manufacturer’s instructions.

### MALDIxin test

The bacterial pellet was resuspended in 200 µL of distilled water, washed three times with double-distilled water, and resuspended in 100 µL of double-distilled water. A 50 µL aliquot was then submitted to mild-acid hydrolysis by adding 50 µL of 2% acetic acid in double-distilled water and heating for 1 hour at 100°C. The hydrolyzed cells were spun, the supernatant was discarded, and the pellet was suspended in 25 µL of double-distilled water. An aliquot of 0.4 µL of the bacterial solution was loaded onto the target and immediately overlaid with 0.8 µL of a super-2,5-dihydroxybenzoic acid matrix (Sigma-Aldrich, Gillingham, UK) used at a final concentration of 10 mg/mL in chloroform/methanol (90:10, vol/vol). The bacterial solution and matrix were mixed directly on the target by pipetting, and the mix was dried gently under a stream of air (<1 min). Using the reflectron mode, MALDI-TOF MS analysis was performed on a 4800 Proteomics Analyzer (Applied Biosystems, Foster City, CA, USA). Samples were analyzed by operating at 20 kV in the negative-ion mode using an extraction delay set at 20 ns. MS data were analyzed using Data Explorer version 4.9 (Applied Biosystems)

### RNA extraction, RNA-seq library construction, and Illumina sequencing

Total bacterial RNAs for RNA-seq and qRT-PCR analysis were isolated from bacterial cultures grown as described above. Three biological replicates were used to represent each sample at 26°C (control) and 37°C, resulting in 24 samples in total and six pairwise comparisons. The cultures were homogenized with 0.1 mm-sized glass beads with a Mini-Beadbeater (Biospec Products, Inc.). TRIzol Reagent (Life Technologies, Inc.) was used to isolate total RNA according to the manufacturer’s instructions, followed by DNase I treatment (Roche). The NanoDrop 1000 (NanoDrop Technologies, Wilmington, DE, USA) and Agilent 2100 Bioanalyzer (Agilent Technologies, Santa Clara, CA, USA) were used to assess RNA concentration and integrity, respectively, of the 24 samples. In addition, the integrity of the RNA samples was further tested in agarose gel before being used for subsequent analysis.

For the library preparation, the bacterial mRNAs were enriched by depleting ribosomal RNA using a Ribo-Zero rRNA removal kit (Illumina). Following depletion, 2.5 µg of total RNA from each sample in duplicate was utilized as the initial material for creating cDNA libraries. The strand-specific cDNA libraries of *Y. pseudotuberculosis* were constructed using the ScriptSeq Complete Bacteria Kit (Illumina) according to the manufacturer’s instructions, while the library concentrations were measured with a Qubit 2.0 Fluorometer (Life Technologies, Inc.). Finally, 10 pmol libraries were used within Illumina Reagent Kit v.3 (150 cycles) and sequenced with the MiSeq System (Illumina, Inc.), and adapters were trimmed by MiSeq internal software.

### Reads processing, mapping, and gene expression quantification

In this study, the reference genome of *Y. pseudotuberculosis* YPIII (NC_010465 for the chromosome and NC_006153 for the pYV plasmid) was used. The CLC-Bio Genomic Workbench (QIAGEN) was then utilized for quality, ambiguity, length trimming, alignment with the reference genome, and normalization of reads per kilobase per million mapped reads. The CLC-Bio Genomic Workbench was used with default parameters for quality, ambiguity, and length trimming. The rRNA and tRNA annotations were deleted from the genome annotation files before mapping to avoid any bias related to rRNA depletion during library preparation. The Q20 percentages were more than 95%, while the Q30 base percentage, which is an indicator of the overall reproducibility and quality of the assay, was greater than 90%. Moreover, the GC contents of all the reads were above 45% for all 24 samples. Differential expression analysis was performed using the DESeq2 R package to identify DEGs between the controls and treated samples. To estimate the expression level, the DESeq2 program was used to normalize the number of counts of each sample gene using the base means. The difference was calculated, and the statistical significance was determined using the negative binomial distribution test (65, 66). Genes with a standard fold change of less than or equal to 1 (≥1 or ≤−1) and a *P*-value of ≤0.05 between control and treated samples were considered differentially expressed.

### Validation of DEGs by qRT-PCR

To validate the reliability and repeatability of the RNA-seq data, six DEGs were randomly selected for verification by qRT-PCR. Notably, the qRT-PCR performed for *ureE*, *wzz*, and *manB* genes was in the *m-ops* background and compared to the wt at 26°C. Similarly, *fliQ* and YPK_1228 were also from the *m-ops* background and compared to the wt, but at 37°C. The qRT-PCR for the *yscD* gene was in the Δ*O-aB* background and compared to the wt at 37°C. The bacterial strains were grown, and the total RNA extracted was used as templates for cDNA synthesis with the RevertAid H Minus First Strand cDNA Synthesis Kit (Fermentas). The gene-specific primers (Table S2) were designed using Primer Premier 5.0 software (Premier Biosoft International, Palo Alto, CA, USA). The qRT-PCRs were performed in triplicate for each condition using KAPA SYBR FAST qPCR Master Mix (KAPA Biosystems) and a Real-Time PCR Detection System (Bio-Rad). The stable reference gene YPK_0340 (*rpoB*) was selected as an internal control to normalize the expression data. The relative expression levels of the six DEGs were calculated according to the 2−ΔΔCT (cycle threshold) method.

## Data Availability

The resulting RNA-seq data files described in this study have been deposited in the Gene Expression Omnibus (GEO) database, at NCBI, under the accession number GSE272323.

## SUPPLEMENTAL MATERIAL

Supplemental figures mbio.02122-25-s0001.pdf Fig. S1 and S2.

Table S1 mbio.02122-25-s0002.docx Strains.

Table S2 mbio.02122-25-s0003.docx Primers of six differentially expressed genes used for qRT-PCR verification.

ASM does not own the copyrights to Supplemental Material that may be linked to, or accessed through, an article. The authors have granted ASM a non-exclusive, world-wide license to publish the Supplemental Material files. Please contact the corresponding author directly for reuse.
