# Supplementary material for: RfaH is essential for virulence and adaptive responses in Yersinia pseudotuberculosis infection
Source: mBio. 2025 Sep 29;16(11):e02122-25. doi: 10.1128/mbio.02122-25 (PMC12607645; doi:10.1128/mbio.02122-25)
Supplement: Supplemental figures — Fig. S1 and S2. [file mbio.02122-25-s0001.pdf]

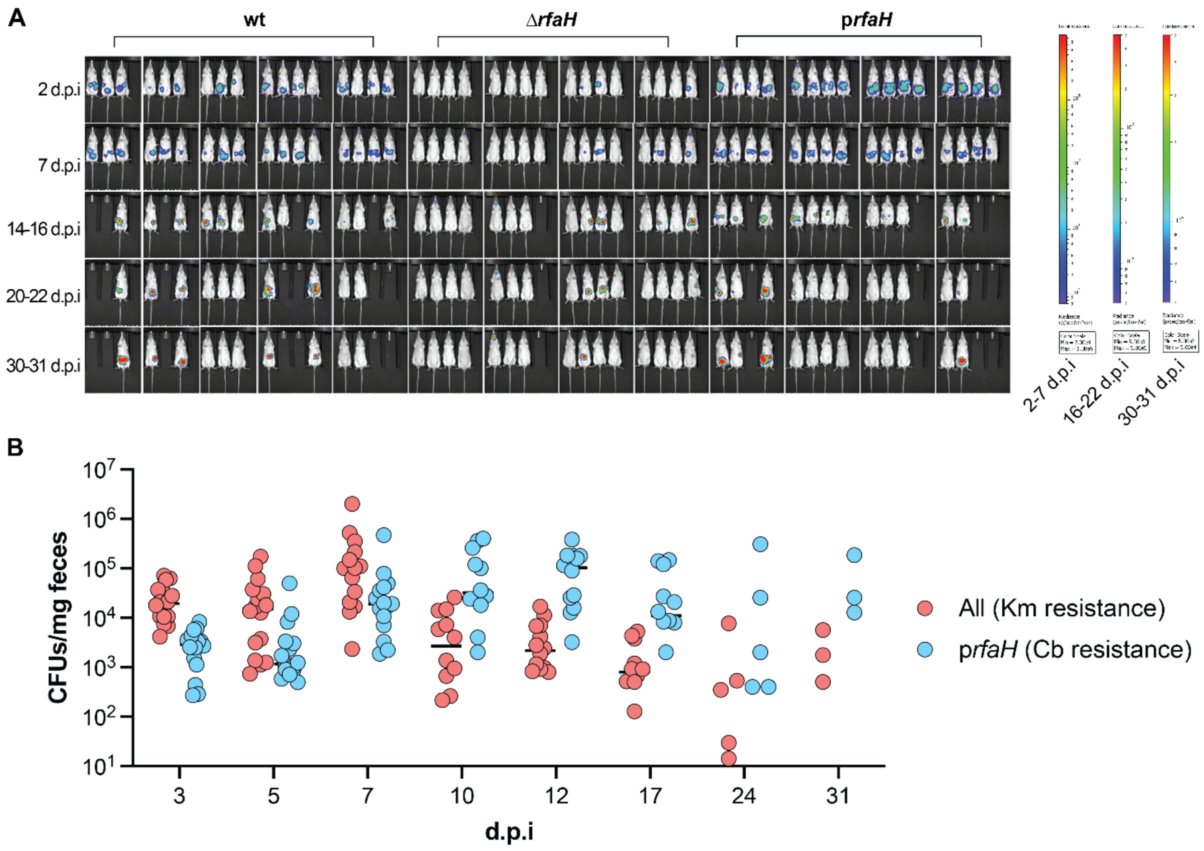

Figure S1. The low dose oral infection of FVB/n mouse leads to virulence attenuation in ΔrfaH and can be complemented with trans expression of rfaH. **(A)** Mice were infected orally with overnight cultures of bacteria. The infection process was monitored by days post-infection (d.p.i) by detecting total photon emission, using the IVIS Spectrum system. The intensity of bioluminescent emission is represented as pseudocolors with variations in color representing light intensity; red represents the most intense light emission, while blue corresponds to the weakest signal. **(B)** The screening of antibiotic resistance was done by monitoring the presence of prfaH during infection. The presence of prfaH was constantly monitored by screening for antibiotic resistance encoded on plasmids encoding rfaH as Y. pseudotuberculosis was shed through feces.

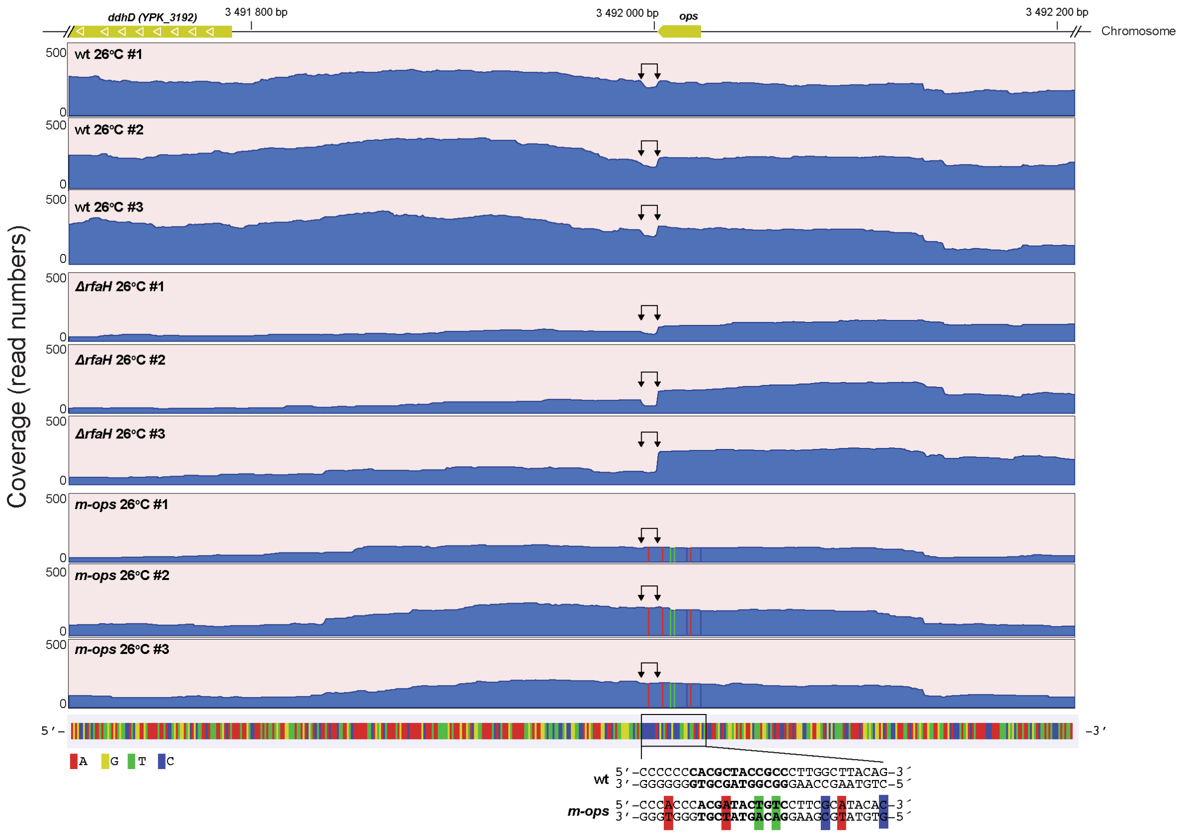

Figure S2. RNA-seq read mappings of the O-antigen biosynthesis operon's 5' UTR indicate the presence of a hypothetical non-coding RNA. Read coverage tracks (blue) for the 5' UTR of the O-antigen biosynthesis operon (upstream of *ddhD*) and the *ops* sequence across triplicates of wt, *ΔrfaH*, and *m-ops* strains at 26°C. The relatively low coverage of the GGGGGGGG motif downstream of the *ops* sequence in the wt and *ΔrfaH* strains is highlighted by two arrowheads in each track. In contrast, this low coverage of the GGGGGGG sequence is absent in the *m-ops* strain, likely due to a point mutation that results in the GGGTGGG sequence. The bottom track displays the DNA sequence with nucleotides represented in distinct colors. The *ops* sequence and its flanking regions in the wt and *m-ops* strains are delineated below, with the *ops* sequence in bold and nucleotide substitutions in the *m-ops* strain indicated by color coding.
