# Supplementary material for: RfaH is essential for virulence and adaptive responses in Yersinia pseudotuberculosis infection
Source: mBio. 2025 Sep 29;16(11):e02122-25. doi: 10.1128/mbio.02122-25 (PMC12607645; doi:10.1128/mbio.02122-25)
Supplement: Table S2 — Primers of six differentially expressed genes used for qRT-PCR verification. [file mbio.02122-25-s0003.docx]

| **Table S2.** The primers of seven differentially expressed genes used for qRT-PCR verification | | | |
| --- | --- | --- | --- |
| **Gene_ID** | **Gene name** | **Primer_F** | **Primer_R** |
| YPK_1134 | ureE | CGTTGCGACCACCATGAT | TGCGTGTCGGTATCCTCA |
| YPK_0705 | *fliQ* | TCAGGCCACCACACAGAT | TACCCAACCAACTGTGCG |
| YPK_1228 |  | AGGGGTTATGACGGCTGT | TCATCAATCAGCGGTGCG |
| YPK_3916 | *yscD* | GGAGACATTGGCCGCTTT | ACTCCCTTGCTGCAAACG |
| YPK_3177 | *wzz* | CTATGGGATCCGATGCGC | TAGTGGGTTCCGTTGGCT |
| YPK_3178 | *manB* | CAATGGTGCTGCTGGTCA | AGTAGTATCCTGGCGGCA |
